# P122: Hand hygiene in Swedish health care – past and present work

**DOI:** 10.1186/2047-2994-2-S1-P122

**Published:** 2013-06-20

**Authors:** O Aspevall

**Affiliations:** 1 Analysis and Prevention, Antibiotics and Infection Control Unit, Swedish Institute for Communicable Disease Control, Solna, Sweden

## Introduction

Hand hygiene (HH) is widely recognized as one of the most effective preventive measures against health care associated infections (HCAI). Alcoholic hand rub (AHR) has been used as standard HH method in Swedish HC for more than three decades. Infection control (IC) experts in Sweden regards this as one among several explanations for the low incidence of antibiotic resistant bacteria, e.g. MRSA (methicillin resistant Staphylococcus aureus), in Swedish HC.

## Objectives

This study describes the development in Sweden regarding implementing HH in HC from the 1980-ties to the present. The aims are to find and present the for the implementation of HH most important professional groups/organizations, events, projects or campaigns, as well as important factors for success.

## Methods

Interview study, key persons with long experience from IC in Sweden were interviewed.

## Results

AHR has been recommended as for HH in Sweden since 1980 [1].

IC within all counties have systematically promoted HH since at least two to three decades. Compliance measurements, AHR consumption measurements, as well as local campaigns have been used.

In 2004 the Swedish Association of Local Authorities and Regions (SALAR) launched a project called VRISS (HCAI should be stopped). This worked with bundles to reduce e.g. urinary tract infections. All bundles included HH measures.

The National Board of Health and Welfare published regulations on basic hygiene including HH in 2007.

All HC participates in prevalence measurements for HCAI since fall 2008. Results are published nationally.

All counties participate in nationally reported compliance measurements since fall 2010. Elderly care provided by municipalities is increasingly joining this as well.

Last year a national working group adapted the WHO SAVE LIVES: Clean Your Hands and published a national package to promote HH. It’s called 'Clean Hands Saves Lives'.

For May 7 an inspirational day for hand hygiene is planned.

## Conclusion

Focus on HH can never be allowed to decline. Despite a long tradition of using AHR in HC, there has recently occurred several outbreaks of resistant bacteria in Swedish hospitals. With renewed attention to HH compliance among other actions these were successfully stopped.

## Disclosure of interest

None declared
